# Computational identification of microRNAs associated to both epithelial to mesenchymal transition and NGAL/MMP-9 pathways in bladder cancer

**DOI:** 10.18632/oncotarget.11805

**Published:** 2016-09-01

**Authors:** Luca Falzone, Saverio Candido, Rossella Salemi, Maria S. Basile, Aurora Scalisi, James A. McCubrey, Francesco Torino, Salvatore S. Signorelli, Maurizio Montella, Massimo Libra

**Affiliations:** ^1^ Department of Biomedical and Biotechnological Sciences, Laboratory of Translational Oncology and Functional Genomics, Section of General and Clinical Pathology and Oncology, University of Catania, Catania, Italy; ^2^ Oncological Pathology Unit, ASP, Catania, Italy; ^3^ Department of Microbiology and Immunology, Brody School of Medicine, East Carolina University, Greenville, NC, USA; ^4^ Department of Systems Medicine, Chair of Medical Oncology, Tor Vergata University of Rome, Rome, Italy; ^5^ Department of Clinical and Experimental Medicine, University of Catania, Medical Angiology Unit, Garibaldi Hospital, Catania, Italy; ^6^ Unit of Epidemiology, ‘Fondazione G. Pascale’, Istituto Nazionale Tumori, Naples, Italy

**Keywords:** bladder cancer, bioinformatics, miRNAs, epithelial-mesenchymal transition, NGAL/MMP-9

## Abstract

Bladder cancer is one of the leading cancer of the urinary tract. It is often diagnosed at advanced stage of the disease. To date, no specific and effective early detection biomarkers are available. Cancer development and progression are associated with the involvement of both epithelial-mesenchymal transition (EMT) and tumor microenvironment of which NGAL/MMP-9 complex represents the main player in bladder cancer. It is known that change in microRNAs (miRNAs) expression may result in gene modulation. Therefore, the identification of specific miRNAs associated with EMT pathway and NGAL/MMP-9 complex may be useful to detect the development of bladder cancer at early stages.

On this ground, the expression levels of miRNAs in public available datasets of bladder cancer containing data of non-coding RNA profiling was evaluated. This analysis revealed a group of 16 miRNAs differentially expressed between bladder cancer patients and related healthy controls. By miRNA prediction tool (mirDIP), the relationship between the identified miRNAs and the EMT genes was established. Using the DIANA-mirPath (v.2) software, miRNAs, able to modulate the expression of NGAL and MMP-9 genes, were recognized.

The results of this study provide evidence that the downregulated hsa-miR-145-5p and hsa-miR-214-3p may modulate the expression of both EMT and NGAL/MMP-9 pathways. Therefore, further validation analyses may confirm the usefulness of these selected miRNAs for predicting the development of bladder cancer at the early stage of the disease.

## INTRODUCTION

Bladder cancer is one of the most common worldwide malignancies. In developed Countries, it is the ninth most frequent cancer and the fourteenth leading cause of death in cancer patient [1]. In urology, bladder cancer is the second most prevalent malignancy after the prostate cancer [2]. According to the GLOBOCAN 2012 Cancer Fact Sheets, in the most developed Countries Bladder cancer incidence is three times higher among men (6.1%) than among women (2.0%). The corresponding mortality rates for men and women are 3.7% and 1.6%, respectively [3].

Several factors contribute to the development of bladder cancer, including tobacco smoking, as the major risk factor [4, 5]. Other risk factors are represented by obesity, hypertension, diabetes, and chronic exposure to aromatic amines and nitrosamines contained in paints and dyes [6–8]. In addition, the development of bladder cancer is promoted by unhealthy food habits and metabolic syndrome [9, 10].

Unfortunately, the percentage of recurrence for this cancer is very high and patients need to be frequently monitored. Although invasive and expansive, cystoscopy remains the most appropriate diagnostic technique used for the diagnosis and follow-up of bladder cancer. Another diagnostic method is the urinary cytology test, but this technique does not seem to have much sensitivity, especially in low-grade bladder cancer, although it is well tolerated by patients [11–13].

The discovery of new markers may be helpful for an early diagnosis and for classifying patients with high risk of cancer recurrence. A growing body of evidence suggests that microRNAs (miRNAs) can be used as markers with high specificity and sensitivity capable of identifying early cancer lesions through the study of the interactions between specific miRNAs with different pathways of tumor aggressiveness. miRNAs are small, noncoding RNA species with a length of 20–22 nucleotides that negatively regulate gene expression interfering with mRNA abundance and transcriptional efficiency [14, 15].

It is well known that in cancer, miRNAs play a key role in regulating the expression of genes associated with carcinogenesis or tumor suppression. Accordingly, changes in miRNAs expression levels may allow the identification of biological alterations, such as epithelial-mesenchymal transition (EMT), which represent a crucial event in cancer progression and aggressiveness [16–19]. For instance, we have recently demonstrated the involvement of NGAL and MMP-9 in bladder cancer development and progression [20]. The role of these proteins in tumor invasion and metastatic dissemination is largely known as they interact with each other forming the NGAL/MMP-9 complex that in turn promotes the extracellular matrix degradation process necessary for tumor spreading [21, 22]. Therefore, it was intriguing to identify which miRNAs may be able to modulate the expression of such proteins in bladder cancer.

On these bases, in the present study we performed a computational analysis to identify which miRNAs were differentially expressed between bladder cancer patients and related healthy controls. Furthermore, among these miRNAs we identified which of them are able to interact with a wide range of genes involved in EMT and NGAL/MMP-9 pathways but also in many other different cancer pathways. The identification of these miRNAs may be useful for the early diagnosis of bladder cancer development and progression.

## RESULTS

### Identification of putative miRNAs of interest in bladder cancer

The differential analysis between bladder cancer patients and related healthy controls revealed 15 different miRNAs that were differentially expressed in both GSE40355 and GSE39093 datasets (*p* < 0.01) (Table 1). Among these, 9 were up-regulated and 6 were down-regulated with similar expression levels in both datasets. Moreover, the differential analysis between high-grade and low-grade bladder cancer revealed that the hsa-miR-18b-5p was the only miRNA differentially expressed in both datasets (*p* < 0.05) (Table 1).

**Table 1:** miRNAs differentially expressed in bladder cancer patients and healthy controls in both GSE40355 and GSE39093 datasets

miRNAs	Fold Change (GSE40355)	Fold Change (GSE39093)
**Cancer vs Normal (*p* < 0.01)**		
** hsa-miR-100-5p**	−80.00*	−2.57*
hsa-miR-106b-5p	3.84	2.25*
hsa-miR-125b-5p	−24.86	−2.54*
hsa-miR-143-3p	−36.39*	−1.97
** hsa-miR-145-5p**	−46.55*	−2.28*
hsa-miR-17-5p	2.79	1.83
** hsa-miR-182-5p**	121.88*	2.87*
hsa-miR-19b-3p	3.39	1.84*
** hsa-miR-200a-3p**	22.09*	2.53*
hsa-miR-200a-5p	603.88*	1.55
hsa-miR-200b-5p	94.15*	1.74
hsa-miR-20a-5p	2.78	2.36*
** hsa-miR-214-3p**	−34.88*	−2.15*
hsa-miR-425-5p	5.83*	1.79
** hsa-miR-99a-5p**	−166.50*	−2.97*
**High vs Low (*p* < 0.05)**		
hsa-miR-18b-5p	17.88	0.65

* Top 10 miRNAs differentially expressed. In bold are reported the miRNAs within the top 10 of both datasets.

Among the 16 miRNAs described in the Table 1, we performed the top 10 list of the most up-regulated or down-regulated miRNAs for each dataset. Then, we merged both top 10 lists of miRNAs of the two datasets and identified the following six miRNAs: hsa-miR-182-5p, hsa-miR-200a-3p, hsa-miR-99a-5p, hsa-miR-100-5p, hsa-miR-145-5p and hsa-miR-214-3p.

### Putative miRNAs involved in epithelial to mesenchymal transition and NGAL/MMP-9 pathways

**Figure 1:**
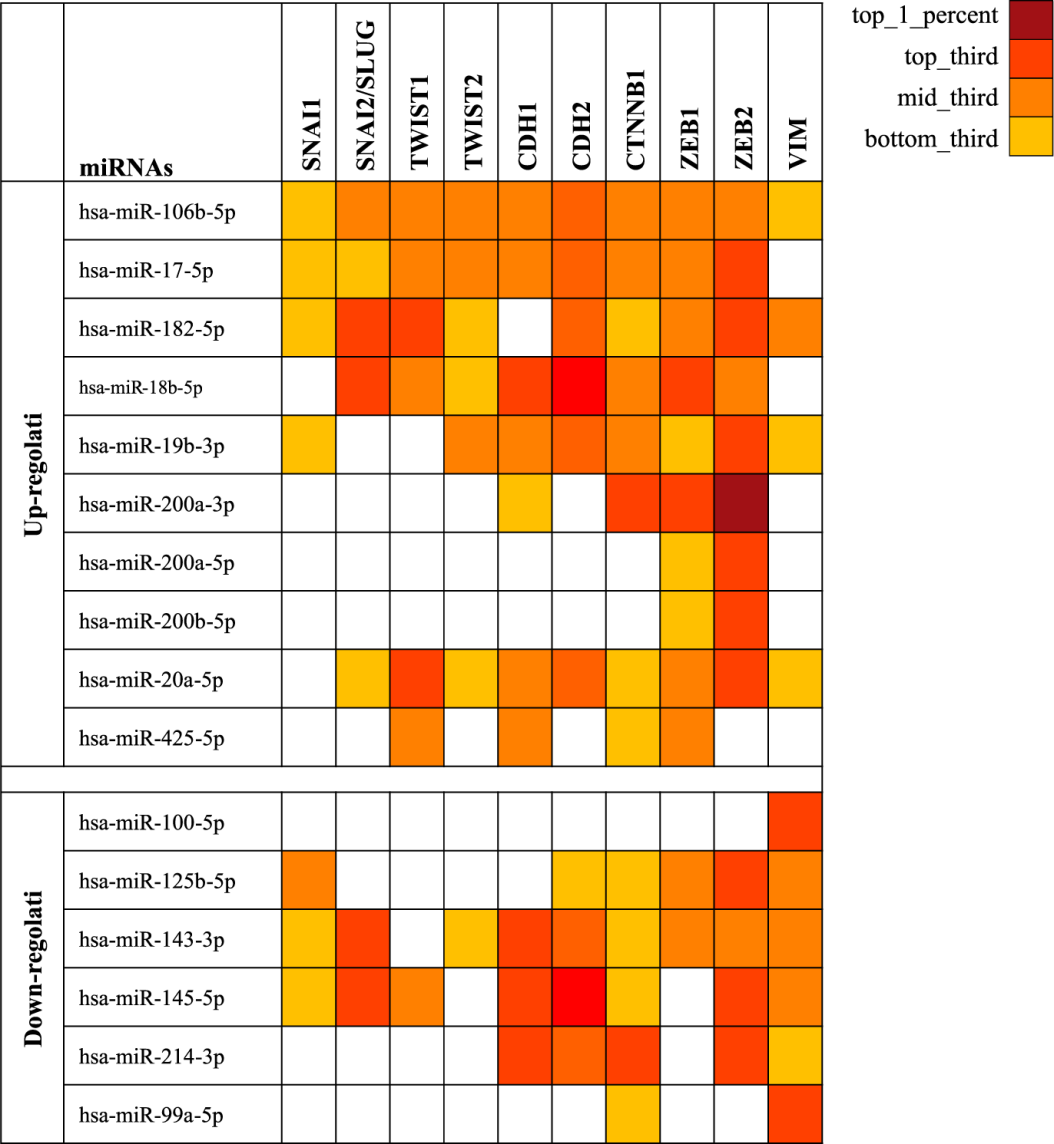
miRNAs able to target the most important genes involved in the ephythelial-mesenchymal transition The gradations of color correspond to the specific values of each miRNA to the target genes. SNAI1: snail family zinc finger 1; SNAI2: snail family zinc finger 2; TWIST1: twist bHLH family transcription factor 1; TWIST2: twist bHLH family transcription factor 2; CDH1: E-cadherin; CTNNB1: β-catenin; ZEB1: zinc finger E-box binding homeobox 1; ZEB2: zinc finger E-box binding homeobox 2; VIM: vimentin.

Prediction analysis performed by mirDIP bioinformatic tool showed how the 16 selected miRNAs are able to target a wide range of genes involved in the EMT. As shown in Figure 1, several miRNAs can alter the expression of multiple EMT genes simultaneously, probably playing a major key role in cancer development and aggressiveness. For each miRNA was also reported the value of specificity against related target genes (Figure 1).

The use of mirDIP software has also allowed us to identify differentially expressed miRNAs that were down-regulated in bladder cancer and therefore responsible for the increase of gene expression of MMP-9 and NGAL in cancer patients.

At first, 452 miRNAs, modulating the NGAL expression, were identified. Among these, the following miRNAs hsa-miR-99a-5p, hsa-miR-100-5p, hsa-miR-145-5p and hsa-miR-214-3p were included in the group of 16 miRNAs listed in the Table 1 and were down-regulated in tumor samples when compared to healthy controls in both GSE40355 and GSE39093 datasets (Table 2).

**Table 2:** miRNAs differentially expressed in tumors compared to healthy controls able to target NGAL gene according microRNA Data Integration Portal (mirDIP)

miRNAs	Source	Score Std	Rank
**Up-regulated**			
hsa-miR-18b-5p	PITA_0_0_ALL	54.16	mid_third
hsa-miR-182-5p	RNA22_v_14May2011	7.64	mid_third
hsa-miR-19b-3p	RNA22_v_14May2011	0.21	bottom_third
**Down-regulated**			
hsa-miR-145-5p	RNA22_v_14May2011	19.42	top_third
hsa-miR-99a-5p	RNA22_v_14May2011	16.32	top_third
hsa-miR-100-5p	RNA22_v_14May2011	6.82	bottom_third
hsa-miR-214-3p	microrna_org_conserved_aug_1020	2.59	bottom_third

Similarly, mirDIP analysis identified 628 miRNAs able to recognize and degrade the MMP-9 mRNA. Among these, only the miRNAs hsa-miR-145-5p, hsa-miR-214-3p and hsa-miR-125b-5p were down regulated in tumor samples when compared to healthy controls in the same two datasets (Table 3). According to this analysis, it is clear that both hsa-miR-214-3p and hsa-miR-145-5p miRNAs may be able to target and modulate MMP-9 and NGAL genes. Therefore, their role in tumor cell invasion and dissemination may be indicated.

**Table 3:** miRNAs differentially expressed in tumors compared to healthy controls able to target MMP-9 gene according microRNA Data Integration Portal (mirDIP)

miRNAs	Source	Score Std	Rank
**Up-regulated**			
hsa-miR-200a	PITA_0_0_ALL	47.67	bottom_third
hsa-miR-141	PITA_0_0_ALL	46.38	bottom_third
hsa-miR-130b	RNA22_v_14May2011	17.98	top_third
hsa-miR-138	RNA22_v_14May2011	16.32	top_third
hsa-miR-934	RNA22_v_14May2011	15.29	top_third
hsa-miR-7	RNA22_v_14May2011	14.05	mid_third
hsa-miR-513b	RNA22_v_14May2011	11.36	mid_third
hsa-miR-16	RNA22_v_14May2011	10.12	mid_third
hsa-miR-200a	RNA22_v_14May2011	7.23	bottom_third
hsa-miR-106b	RNA22_v_14May2011	6.40	bottom_third
hsa-miR-182	RNA22_v_14May2011	5.99	bottom_third
hsa-miR-141	RNA22_v_14May2011	3.72	bottom_third
**Down-regulated**			
hsa-miR-145	RNA22_v_14May2011	22.11	top_third
hsa-miR-214	RNA22_v_14May2011	21.28	top_third
hsa-miR-195	RNA22_v_14May2011	13.22	mid_third
hsa-miR-125b	RNA22_v_14May2011	7.23	bottom_third

### Identified miRNAs in the main pathways in cancer

To understand the role of miRNAs in cancer development, the DIANA-mirPath analysis of the six selected highly-modulated miRNAs in both bladder cancer datasets was performed. The data showed that the miRNAs hsa-miR-182-5p, hsa-miR-200a-3p, hsa-miR-99a-5p, hsa-miR-100-5p, hsa-miR-145-5p, hsa-miR-214-3p may alter the transcriptional levels of several genes grouped in different pathways such as bladder cancer pathway (has05219), adherens junction pathway (hsa04520), PIK3CA-Akt signal pathway (hsa04151), MAPK signal pathway (hsa04010) and in general cancer pathways (hsa05200) (Table 4).

**Table 4:** Interaction between selected miRNAs and target genes in different pathways (DIANA-mirPath v.2)

miRNAs	*p* Value	Gene Target
**Pathways in cancer (hsa05200)**		
hsa-miR-200a-3p	0.006320485	TCF7L1, CTNNB1
hsa-miR-182-5p	0.01027914	MITF, EP300, CDKN1A, FOXO1
hsa-miR-99a-5p	0.006069299	FGFR3, MTOR
hsa-miR-145-5p	0.000288274	IGF1R, TPM3, MMP1, MYC, CDKN1A, STAT1
hsa-miR-214-3p	0.03587201	TP53, MAPK8, DAPK1, PTEN
**Bladder cancer (hsa05219)**		
hsa-miR-99a-5p	0.005785765	FGFR3
hsa-miR-100-5p	0.005422842	FGFR3
hsa-miR-145-5p	0.000288274	MMP1, MYC, CDKN1A
hsa-miR-214-3p	0.00565335	TP53, DAPK1
**Adherens junction (hsa04520)**		
hsa-miR-200a-3p	1.30E–06	TCF7L1, WASF3, CTNNB1
**PI3K-Akt signaling pathway (hsa04151)**		
hsa-miR-99a-5p	0.005785765	FGFR3, MTOR
hsa-miR-100-5p	0.04890642	FGFR3
**MAPK signaling pathway (hsa04010)**		
hsa-miR-100-5p	0.03917171	FGFR3
hsa-miR-214-3p	0.002472282	MAP2K5, MAP2K3, TP53, RASA1, MAPK8

## DISCUSSION

Recently, the discovery of extracellular miRNAs, relatively stable in serum, plasma and urine has generated a considerable interest, since the variations in their expression levels can be used as non-invasive indicators for different kinds of diseases, including cancer. Currently, no specific markers are available for the early detection of bladder cancer. In this context, the analysis of specific miRNAs may represent a good model for the early diagnosis and follow-up in different cancer types, including bladder cancer [23–25].

A growing number of studies analyzed circulating miRNAs in different tumor types including those of bladder. However, their role in cancer development or progression was not deeply clarified. To our knowledge, this is the first computational study analyzing the putative miRNA interacting with oncogenic pathways involved in bladder cancer.

First, the analysis was conducted on non-coding RNA profiling by array datasets to identify putative miRNAs differentially expressed in bladder cancer patients and healthy controls. This preliminary bioinformatics approach allowed us to identify six different miRNAs involved in bladder cancer (hsa-miR-182-5p, hsa-miR-200a-3p, hsa-miR-99a-5p, hsa-miR-100-5p, hsa-miR-145-5p and hsa-miR-214-3p).

We recently observed that NGAL/MMP-9 complex plays an active role in proliferation, differentiation, tissue development, and tumor development of bladder cancer progression [21]. Of note, NGAL and MMP-9 are molecules that are found in the tumor microenvironment. In particular, NGAL regulates the expression of MMP-9, which is responsible for several physiological and pathological processes including the enzymatic remodeling of the extracellular matrix during angiogenesis, tumor growth and metastasis. These two proteins form the MMP-9/NGAL complex capable of protecting MMP-9 from proteolytic degradation and hence favoring the tumor invasiveness. These mechanisms are supported by our previous computational analysis, performed by “The Human Protein Atlas” (http://www.proteinatlas.org/), which suggested an active role of NGAL, in cancer development in different tumor histotypes [26]. Accordingly, the result of the present study show that the down-regulated hsa-miR-145-5p and hsa-miR-214-3p are able to modulate MMP-9 and NGAL genes and also some genes of EMT pathway. In particular, the epigenetic modification of EMT pathway made by the miRNAs may represent a fundamental step in malignance transformation of tumor cells. Several studies support the idea that miRNAs are strictly involved in the regulation of epithelial-to-mesenchymal transition in which tumor cells tumor cells acquire new mesenchymal characteristics and lose their original epithelial features [27, 28]. The results of this transformation are the increase of tumor aggressiveness, of metastatic lesions and the development of resistance to chemo- immunotherapies [29, 30].

In addition, DIANA-mirPath analysis has shown that the six selected miRNAs have a fundamental role in different cancer pathways highlighting their possible involvement in bladder cancer. The bioinformatics tool revealed that the miRNAs identified are able to target several genes, such as FGFR3, MTOR, FOXO1, MYC, TP53, STAT1 etc., known to be involved in cancer onset and development [31–35]. The modulation of these cancer pathways and genes by the miRNA induces the increase of cell proliferation and inhibition of tumor cell apoptosis leading to tumor spreading.

Furthermore, the detection of these putative miRNAs for the early diagnosis of cancer can be very effective in bladder because the analysis can be extended not only in serum or plasma samples, but also in urine samples where these miRNAs may be directly released by tumor cells, even in the early stages of the disease [36, 37].

Further studies will be needed to determine the levels of sensitivity and specificity of the putative miRNAs identified in this study in predicting the development of bladder cancer. In particular, the analysis will be conducted on a wide range of patients with bladder cancer and related healthy controls. The analysis will be conducted to determine the expression levels of hsa-miR-145-5p and hsa-miR-214-3p, able to modulate MMP-9 and NGAL expression levels. In addition, the analysis of hsa-miR-182-5p, hsa-miR-200a-3p, hsa-miR-99a-5p, hsa-miR-100-5p will be performed because these miRNAs were found to be the most differentially expressed between bladder cancer patients and related healthy controls in both GSE40355 and GSE39093 datasets.

Finally, the study on specific miRNAs differentially expressed in bladder cancer patients could have a great clinical implication because miRNAs are detectable not only in tissue samples but also in several biological fluids, such as in plasma and urine, where circulating tumor cells (CTCs) release them. According to these hypothesis, the identification of changes in the expression levels of individual or multiple miRNAs directly into peripheral blood, or in urine, may represent a new strategy for the early detection of cancer and for the prediction of cancer recurrence development [38–41].

## MATERIALS AND METHODS

### miRNAs computational analysis

In order to identify putative miRNAs that were differentially expressed between bladder cancer patients and related healthy controls, datasets of non-coding RNA profiling available on Gene Expression Omnibus (GEO) Dataset (www.ncbi.nlm.nih.gov/geo/) were analyzed. Among all publicly available GEO datasets, only those containing bladder cancer samples with miRNAs expression levels, clinical data and relative normal controls were analyzed. According to these criteria only the datasets GSE40355 and GSE39093 were used for our analysis [42, 43].

The GSE40355 dataset (Agilent-019118 Human miRNA Microarray 2.0 G4470B [miRNA ID version]) includes 8 samples of high-grade papillary urothelial carcinoma, 8 low-grade papillary urothelial carcinoma and 8 samples of nonmalignant bladder. For all 24 samples clinical data were available. The GSE39093 dataset ([miRNA-1_0] Affymetrix miRNA Array) includes 5 samples of non-muscle invasive bladder cancer (NMIBC) and 5 corresponding adjacent normal tissue, and 5 samples of muscle invasive bladder cancer (MIBC) and 5 corresponding adjacent normal tissue. For all 20 samples clinical data were available.

Differential analysis of miRNAs expression levels (fold change value) between tumor and normal samples was performed in both datasets. Furthermore, the differential analysis between high-grade and low-grade bladder cancer was performed in the same manner.

Finally was performed the top 10 list of the most up-regulated or down-regulated miRNAs for each dataset. This analysis allowed us to identify the 10 most up-regulated and down-regulated miRNAs in bladder cancer compared to healthy controls. Subsequently, among these, were selected the 6 miRNAs in common and strongly modulated in both datasets.

### Interaction between selected miRNAs and epithelial-mesenchimal transition pathway

Through the use of the bioinformatic prediction tool microRNA Data Integration Portal (mirDIP) (http://ophid.utoronto.ca/mirDIP), was evaluated the interaction between the miRNA previously identified by computational analysis and the main genes involved in EMT [44]. This bioinformatics software integrates twelve microRNA prediction datasets from six different microRNA prediction databases providing a list of EMT gene targeting the specific miRNAs previously indentified.

These genes are SNAI1: snail family zinc finger 1; SNAI2: snail family zinc finger 2; TWIST1: twist family bHLH transcription factor 1; TWIST2: twist family bHLH transcription factor 2; CDH1: E-caderina; CTNNB1: β-catenina; ZEB1: zinc finger E-box binding homeobox 1; ZEB2: zinc finger E-box binding homeobox 2; VIM: vimentina.

Subsequently we used the mirDIP software to predict which selected miRNAs are able to target the MMP-9 and NGAL genes of which our research group is already in possession of serum and urine expression levels [20].

### Interaction between selected miRNAs and main pathways in cancer

miRNAs are not only involved in the epithelial-mesenchymal transition, but are able to interact with a wide range of genes involved in different cancer pathways. Through the use of the bioinformatic tool DIANA-mirPath (v.2) the top ten differentially expressed miRNA were analyzed to predict miRNA targets in 3′-UTR gene regions according to experimentally validated miRNA interactions derived from DIANA-TarBase v6.0 algorithm. These interactions (predicted and/or validated) were subsequently combined with sophisticated merging and meta-analysis algorithms by DIANA-mirPath.

### Statistical analysis

Statistical analyses and Student's *t*-Tests were performed to select the differentially expressed miRNAs as previously described [45]. No additional normalization procedures were applied to data obtained from GSE40355 and GSE39093 datasets included in this study.

## References

[1] Mahdavifar N, Ghoncheh M, Pakzad R, Momenimovahed Z, Salehiniya H (2016). Epidemiology, Incidence and Mortality of Bladder Cancer and their Relationship with the Development Index in the World. Asian Pac J Cancer Prev.

[2] Malats N, Real FX (2015). Epidemiology of Bladder Cancer. Hematology/oncology clinics of North America.

[3] Ferlay J, Soerjomataram I, Ervik M, Dikshit R, Eser S, Mathers C, Rebelo M, Parkin DM, Forman D, Bray F (2013). GLOBOCAN 2012 v1.0, Cancer Incidence and Mortality Worldwide: IARC CancerBase No. 11 [Internet]. http://globocan.iarc.fr,%20accessed%20on%20day/month/year.

[4] Lee HW, Wang HT, Weng MW, Hu Y, Chen WS, Chou D, Liu Y, Donin N, Huang WC, Lepor H, Wu XR, Wang H, Beland FA (2014). Acrolein- and 4-Aminobiphenyl-DNA adducts in human bladder mucosa and tumor tissue and their mutagenicity in human urothelial cells. Oncotarget.

[5] Brait M, Munari E, LeBron C, Noordhuis MG, Begum S, Michailidi C, Gonzalez-Roibon N, Maldonado L, Sen T, Guerrero-Preston R, Cope L, Parrella P, Fazio VM (2013). Genome-wide methylation profiling and the PI3K-AKT pathway analysis associated with smoking in urothelial cell carcinoma. Cell Cycle.

[6] Xu X, Zhou L, Miao R, Chen W, Zhou Y, Pang Q, Qu K, Liu C (2016). Association of cancer mortality with postdiagnosis overweight and obesity using body mass index. Oncotarget.

[7] Cantiello F, Cicione A, Salonia A, Autorino R, De Nunzio C, Briganti A, Gandaglia G, Dell'Oglio P, Capogrosso P, Damiano R (2015). Association between metabolic syndrome, obesity, diabetes mellitus and oncological outcomes of bladder cancer: A systematic review. Int J Urol.

[8] Goossens ME, Zeegers MP, Bazelier MT, De Bruin ML, Buntinx F, de Vries F (2015). Risk of bladder cancer in patients with diabetes: a retrospective cohort study. BMJ Open.

[9] Montella M, Di Maso M, Crispo A, Grimaldi M, Bosetti C, Turati F, Giudice A, Libra M, Serraino D, La Vecchia C, Tambaro R, Cavalcanti E, Ciliberto G (2015). Metabolic syndrome and the risk of urothelial carcinoma of the bladder: a case-control study. BMC Cancer.

[10] Turati F, Bosetti C, Polesel J, Zucchetto A, Serraino D, Montella M, Libra M, Galfano A, La Vecchia C, Tavani A (2015). Coffee, Tea, Cola, and Bladder Cancer Risk: Dose and Time Relationships. Urology.

[11] Soubra A, Risk MC (2015). Diagnostics techniques in nonmuscle invasive bladder cancer. Indian J Urol.

[12] Mossanen M, Gore JL (2014). The burden of bladder cancer care: direct and indirect costs. Curr Opin Urol.

[13] James AC, Gore JL (2013). The costs of non-muscle invasive bladder cancer. Urol Clin North Am.

[14] Hafsi S, Candido S, Maestro R, Falzone L, Soua Z, Bonavida B, Spandidos DA, Libra M (2016). Correlation between the overexpression of Yin Yang 1 and the expression levels of miRNAs in Burkitt's lymphoma: A computational study. Oncol Lett.

[15] Leonardi GC, Candido S, Carbone M, Colaianni V, Garozzo SF, Cinà D, Libra M (2012). microRNAs and thyroid cancer: biological and clinical significance (Review). Int J Mol Med.

[16] Braicu C, Cojocneanu-Petric R, Chira S, Truta A, Floares A, Petrut B, Achimas-Cadariu P, Berindan-Neagoe I (2015). Clinical and pathological implications of miRNA in bladder cancer. Int J Nanomedicine.

[17] Martínez-Fernández M, Dueñas M, Feber A, Segovia C, García-Escudero R, Rubio C, López-Calderón FF, Díaz-García C, Villacampa F, Duarte J (2015). A Polycomb-mir200 loop regulates clinical outcome in bladder cancer. Oncotarget.

[18] Ouyang H, Zhou Y, Zhang L, Shen G (2015). Diagnostic Value of MicroRNAs for Urologic Cancers: A Systematic Review and Meta-Analysis. Medicine (Baltimore).

[19] Bagnoli M, De Cecco L, Granata A, Nicoletti R, Marchesi E, Alberti P, Valeri B, Libra M, Barbareschi M, Raspagliesi F, Mezzanzanica D, Canevari S (2015). Identification of a chrXq27.3 microRNA cluster associated with early relapse in advanced stage ovarian cancer patients. Oncotarget.

[20] Candido S, Di Maso M, Serraino D, McCubrey JA, Bortolus R, Zanin M, Battiston M, Salemi R, Libra M, Polesel J (2016). Diagnostic value of neutrophil gelatinase-associated lipocalin/matrix metalloproteinase-9 pathway in transitional cell carcinoma of the bladder. Tumour Biol.

[21] Candido S, Abrams SL, Steelman LS, Lertpiriyapong K, Fitzgerald TL, Martelli AM, Cocco L, Montalto G, Cervello M, Polesel J, Libra M, McCubrey JA (2016). Roles of NGAL and MMP-9 in the tumor microenvironment and sensitivity to targeted therapy. Biochim Biophys Acta.

[22] Ricci S, Bruzzese D, DI Carlo A (2015). Evaluation of MMP-2, MMP-9, TIMP-1, TIMP-2, NGAL and MMP-9/NGAL complex in urine and sera from patients with bladder cancer. Oncol Lett.

[23] Kim M, Chen X, Chin LJ, Paranjape T, Speed WC, Kidd KK, Zhao H, Weidhaas JB, Slack FJ (2014). Extensive sequence variation in the 3′ untranslated region of the KRAS gene in lung and ovarian cancer cases. Cell Cycle.

[24] Anastasiadis A, de Reijke TM (2012). Best practice in the treatment of nonmuscle invasive bladder cancer. Ther Adv Urol.

[25] Aoki S, Yamada Y, Nakamura K, Taki T, Tobiume M, Honda N (2006). Thymidine phosphorylase expression as a prognostic marker for predicting recurrence in primary superficial bladder cancer. Oncol Rep.

[26] Candido S, Maestro R, Polesel J, Catania A, Maira F, Signorelli SS, McCubrey JA, Libra M (2014). Roles of neutrophil gelatinase-associated lipocalin (NGAL) in human cancer. Oncotarget.

[27] Zaravinos A (2015). The Regulatory Role of MicroRNAs in EMT and Cancer. J Oncol.

[28] Chan SH, Wang LH (2015). Regulation of cancer metastasis by microRNAs. J Biomed Sci.

[29] Zhang P, Sun Y, Ma L (2015). ZEB1: at the crossroads of epithelial-mesenchymal transition, metastasis and therapy resistance. Cell Cycle.

[30] Bonavida B, Jazirehi A, Vega MI, Huerta-Yepez S, Baritaki S (2013). Roles Each of Snail, Yin Yang 1 and RKIP in the Regulation of Tumor Cells Chemo-immuno-resistance to Apoptosis. For Immunopathol Dis Therap.

[31] Jiang G, Wu AD, Huang C, Gu J, Zhang L, Huang H, Liao X, Li J, Zhang D, Zeng X, Jin H, Huang H, Huang C (2016). Isorhapontigenin (ISO) Inhibits Invasive Bladder Cancer Formation *In Vivo* and Human Bladder Cancer Invasion *In Vitro* by Targeting STAT1/FOXO1 Axis. Cancer Prev Res (Phila).

[32] Zhang X, Zhang Y (2015). Bladder Cancer and Genetic Mutations. Cell Biochem Biophys.

[33] Ai X, Jia ZM, Wang J, DI GP, Zhang XU, Sun F, Zang T, Liao X (2015). Bioinformatics analysis of the target gene of fibroblast growth factor receptor 3 in bladder cancer and associated molecular mechanisms. Oncol Lett.

[34] Zhao Y, Qi JG, Yang N, Lin YL, Liang J, Zhu X (2015). Association between MYC rs9642880[T] allele and bladder cancer risk: a meta-analysis. Genet Mol Res.

[35] Duperret EK, Oh SJ, McNeal A, Prouty SM, Ridky TW (2014). Activating FGFR3 mutations cause mild hyperplasia in human skin, but are insufficient to drive benign or malignant skin tumors. Cell Cycle.

[36] Sapre N, Macintyre G, Clarkson M, Naeem H, Cmero M, Kowalczyk A, Anderson PD, Costello AJ, Corcoran NM, Hovens CM (2016). A urinary microRNA signature can predict the presence of bladder urothelial carcinoma in patients undergoing surveillance. Br J Cancer.

[37] Ding M, Li Y, Wang H, Lv Y, Liang J, Wang J, Li C (2015). Diagnostic value of urinary microRNAs as non-invasive biomarkers for bladder cancer: a meta-analysis. Int J Clin Exp Med.

[38] Mirzaei H, Gholamin S, Shahidsales S, Sahebkar A, Jaafari MR, Mirzaei HR, Hassanian SM, Avan A (2016). MicroRNAs as potential diagnostic and prognostic biomarkers in melanoma. Eur J Cancer.

[39] Chang PY, Chen CC, Chang YS, Tsai WS, You JF, Lin GP, Chen TW, Chen JS, Chan EC (2016). MicroRNA-223 and microRNA-92a in stool and plasma samples act as complementary biomarkers to increase colorectal cancer detection. Oncotarget.

[40] Brunetti O, Russo A, Scarpa A, Santini D, Reni M, Bittoni A, Azzariti A, Aprile G, Delcuratolo S, Signorile M, Gnoni A, Palermo L, Lorusso V (2015). MicroRNA in pancreatic adenocarcinoma: predictive/prognostic biomarkers or therapeutic targets?. Oncotarget.

[41] Frères P, Wenric S, Boukerroucha M, Fasquelle C, Thiry J, Bovy N, Struman I, Geurts P, Collignon J, Schroeder H, Kridelka F, Lifrange E, Jossa V (2016). Circulating microRNA-based screening tool for breast cancer. Oncotarget.

[42] Xu C, Zeng Q, Xu W, Jiao L, Chen Y, Zhang Z, Wu C, Jin T, Pan A, Wei R, Yang B, Sun Y (2013). miRNA-100 inhibits human bladder urothelial carcinogenesis by directly targeting mTOR. Mol Cancer Ther.

[43] Hecker N, Stephan C, Mollenkopf HJ, Jung K, Preissner R, Meyer HA (2013). A new algorithm for integrated analysis of miRNA-mRNA interactions based on individual classification reveals insights into bladder cancer. PLoS One.

[44] Shirdel EA, Xie W, Mak TW, Jurisica I (2011). NAViGaTing the Micronome. Using Multiple MicroRNA Prediction Databases to Identify Signalling Pathway-Associated MicroRNAs. PLoS ONE.

[45] Polesel J, Franceschi S, Talamini R, Negri E, Barzan L, Montella M, Libra M, Vaccher E, Franchin G, La Vecchia C, Serraino D (2011). Tobacco smoking, alcohol drinking, and the risk of different histological types of nasopharyngeal cancer in a low-risk population. Oral Oncol.

